# Epidermal Growth Factor Enhances Cellular Uptake of Polystyrene Nanoparticles by Clathrin-Mediated Endocytosis

**DOI:** 10.3390/ijms18061301

**Published:** 2017-06-19

**Authors:** Le Thi Minh Phuc, Akiyoshi Taniguchi

**Affiliations:** 1 Cellular Functional Nanomaterials Group, Research Center for Functional Materials, National Institute for Materials Science, 1-1 Namiki, Tsukuba, Ibaraki 305-0044, Japan; lethiminh.phuc@nims.go.jp; 2 Graduate School of Advanced Science and Engineering, Waseda University, 3-4-1 Okubo, Shinjuku-ku, Tokyo 169-8555, Japan

**Keywords:** EGF, PS NPs, cellular uptake, clathrin-mediated endocytosis

## Abstract

The interaction between nanoparticles and cells has been studied extensively, but most research has focused on the effect of various nanoparticle characteristics, such as size, morphology, and surface charge, on the cellular uptake of nanoparticles. In contrast, there have been very few studies to assess the influence of cellular factors, such as growth factor responses, on the cellular uptake efficiency of nanoparticles. The aim of this study was to clarify the effects of epidermal growth factor (EGF) on the uptake efficiency of polystyrene nanoparticles (PS NPs) by A431 cells, a human carcinoma epithelial cell line. The results showed that EGF enhanced the uptake efficiency of A431 cells for PS NPs. In addition, inhibition and localization studies of PS NPs and EGF receptors (EGFRs) indicated that cellular uptake of PS NPs is related to the binding of EGF–EGFR complex and PS NPs. Different pathways are used to enter the cells depending on the presence or absence of EGF. In the presence of EGF, cellular uptake of PS NPs is via clathrin-mediated endocytosis, whereas, in the absence of EGF, uptake of PS NPs does not involve clathrin-mediated endocytosis. Our findings indicate that EGF enhances cellular uptake of PS NPs by clathrin-mediated endocytosis. This result could be important for developing safe nanoparticles and their safe use in medical applications.

## 1. Introduction

Polystyrene nanoparticles (PS NPs) are widely used as a model for studying the interaction between NPs and cells due to their many advantages, including commercial availability, high quality, diverse sizes and shapes, biocompatibility, biological non-toxicity, and high functionality due to the presence of many chemical groups [1,2,3]. PS NPs are utilized in various research and commercial applications, including biosensors [4], photonics [5], self-assembling nanostructures [6], and spray and exterior paints [7]. The increasingly wide use of PS NPs is leading to a corresponding rise in exposure levels, thereby raising the problem of potential risk to human health. It is therefore essential to investigate potential interactions between PS NPs and cells.

Most PS NPs are less than 100 nm in diameter and could potentially enter mammalian cells [2]. Indeed, PS NPs have been reported to internalize in many types of cells, such as hepatocyte [8], macrophage [9], lung [10], and epithelium [11]. Previous studies showed that PS NPs can enter cells by several pathways, including phagocytosis, clathrin-coated vesicles, caveolae-mediated endocytosis, and macropinocytosis [12,13,14]. The internalization of NPs is affected by a variety of NP factors, such as target dose, particle size, cell type, NP morphology and surface chemistry, NP geometry, and also by cellular responses, such as the cell cycle phase [15,16,17,18,19,20]. However, few studies have assessed the potentially important effects of cellular responses on the uptake efficiency of PS NPs.

Increased synthesis of epidermal growth factor (EGF) is induced by several cellular responses, including epithelial cell growth. The aim of this study was to clarify how EGF affects the uptake by A431 human carcinoma epithelial cells of PS NPs. EGF is widely used for studying cellular responses and acts by binding to its specific receptor, epidermal growth factor receptor (EGFR), on the cell surface [21,22]. The A431 cell line expresses high levels of EGF receptors on the cell surface (2–3 × 10^6^ receptors/cell) [23,24], and thus their adaptation with EGF could lead to changes in the cellular uptake ratio and pathway of PS NPs. Our results indicate that EGF enhances the uptake efficiency of PS NPs by clathrin-mediated endocytosis, inducing the aggregation of EGF receptors on the cell membrane surface. These results could be important for developing safe NPs and their safe use in medical applications.

## 2. Materials and Methods

### 2.1. Cell Culture

The A431 cell line (JCRB Bank, Osaka, Japan) was cultured at 37 °C, 5% CO_2_, in high glucose Dulbecco’s modified Eagle medium (DMEM, Nacalai Tesque, Kyoto, Japan) supplemented with 10% (*v*/*v*) fetal bovine serum (FBS, Biowest, Tokyo, Japan), 100 µg/mL penicillin, and 10 µg/mL streptomycin. The cells were subcultured every 2 days when confluency reached 70–80%.

### 2.2. Characteristics of PS NPs

Fluoresbrite^®^ YG Microspheres (0.05 µm) were bought from Polysciences (Warrington, PA, USA). Their size distribution and stability were analyzed using an electronic light scattering (ELS) detector (ELSZ-2000 ELS, zeta potential, and particle size analyzer, Otsuka Electronics, Osaka, Japan). The results confirmed that the PS NPs were 44.9 ± 10.5 nm in diameter, with a low polydispersity index of 0.053 and a zeta potential of −46.68 mV in culture medium including 10% FBS.

### 2.3. Cellular Uptake of NPs and Inhibition Study

The cellular percent uptake of PS NPs by A431 cells was calculated from the fluorescence intensity of cells containing fluorescently labeled PS NPs. Briefly, A431 cells were seeded at a density 5 × 10^5^ cells/mL in a 6-well plate and incubated at 37 °C, 5% CO_2_, for 24 h. Next, the cells were incubated in medium containing 10 µg/mL NPs with or without EGF (100 ng/mL) for 24 h. Cells untreated with either NPs or EGF were used as control. The cells were washed twice with phosphate buffered saline (PBS) 1× to completely remove excess NPs. The harvested cells were treated with trypsin/EDTA for 12 min, centrifuged at 1200 rpm for 3 min, and then the cell debris was suspended in 1 mL PBS 1×. Cellular uptake was measured using a SP6800 Spectral Analyzer (Sony Biotechnology Inc., Tokyo, Japan).

Inhibition studies were performed by washing the cells twice with PBS and pretreating with 0.45 M sucrose in serum-free medium for 1 h at 37 °C, then medium containing 10 µg/mL nanoparticles with or without 100 ng/mL EGF was added and the cells were incubated for 24 h at 37 °C in the presence of 0.45 M sucrose. The effect of anti-EGFR antibody on EGF-dependent cellular uptake enhancement was determined by exposing cells to 20 ng/mL anti-EGFR antibody either alone or in combination with 100 ng/mL EGF for 24 h at 37 °C. Finally, the A431 cells were washed, harvested, and assessed as described above for determining cellular uptake of NPs.

### 2.4. Distribution of EGF Receptors on the Cell Membrane Surface

The distribution of EGF receptors (EGFRs) on the cell membrane surface was determined using fluorescently labeled anti-EGFR antibody. First, A431 cells were plated in a CELLview cell culture dish (Greiner Bio-one North America Inc., Carolina, NC, USA) at a density 2.5 × 10^4^ cells per compartment for 24 h. Then, the cells were washed with PBS X1, fixed with 4% formaldehyde for 10 min at 37 °C, permeabilized with 0.1% Triton X-100 for 5 min, and then blocked with 1% BSA/10% normal goat serum/0.3 M glycine in 0.1% PBS-Tween for 1 h. The cells were then incubated overnight at 4 °C with fluorescently labeled anti-EGFR antibody (Abcam, London, UK). Nuclear DNA was labeled with DAPI (4′,6-diamidino-2-phenylindole) (Thermo Fisher Scientific, Waltham, MA, USA) and images were obtained with a confocal laser-scanning microscope (LSM510 META, Carl Zeiss Inc., Jena, Germany).

### 2.5. Statistics Analysis

All data were assessed for statistical significance using a statistical software program (Microcal Origin 8.1, Microcal Software Inc., Northampton, MA, USA). The differences between the mean values of different groups were determined using one-way ANOVA with Tukey test. All values are presented as mean ± S.D. with at least 3 independent replicates (*n* ≥ 3). * *p* ≤ 0.05, ** *p* ≤ 0.01, *** *p* ≤ 0.001, as indicated in the figure legends.

## 3. Results

### 3.1. Dose-Dependent Cellular Uptake of PS NPs by A431 Cells

**Figure 1:**
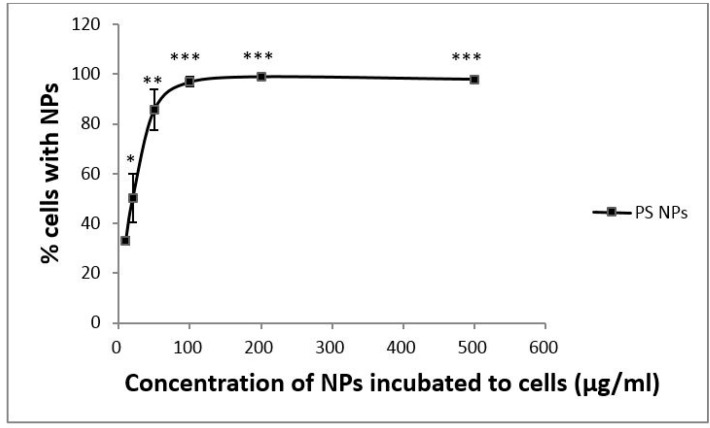
Dose dependency of PS NP cellular uptake by A431 cells. A431 cells were treated with different concentrations of PS NPs (10, 20, 50, 100, 200 and 500 µg/mL) at 37 °C for 24 h. The cellular uptake efficiency of NPs was normalized to that of untreated control cells. Mean values ± standard deviation, *n* = 3. * *p* ≤ 0.05, ** *p* ≤ 0.01, *** *p* ≤ 0.001 compared to each normalized control.

To determine the influence of NP dosage on uptake efficiency, A431 cells were exposed to six concentrations of PS NPs (10, 20, 50, 100, 200 and 500 µg/mL) at 37 °C for 24 h. As shown in Figure 1, uptake efficiency increased in a dose-dependent manner similar to the Michaelis–Menten equation from 0 to 100 µg/mL PS NPs, and then plateaued at concentrations ≥100 µg/mL PS NPs. These results suggested that this concentration (10 µg/mL; 1.5 × 10^11^ NPs/mL) was a good compromise between an observable effect and a condition far to saturation.

### 3.2. EGF Enhanced Cellular Uptake of PS NPs

**Figure 2:**
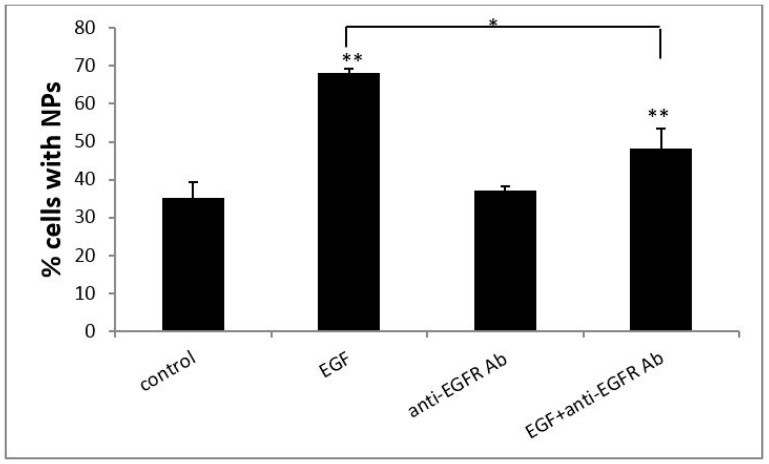
EGF enhanced PS NP cellular uptake efficiency by A431 cells. A431 cells were incubated with 100 ng/mL of EGF (second bar), 20 ng/mL of anti-EGFR antibody (third bar), or 100 ng/mL of EGF and 20 ng/mL of anti-EGFR antibody (fourth bar) at 37 °C for 24 h. The cellular uptake efficiency of NPs was normalized to that of untreated control cells (first bar). Mean values ± standard deviation, *n* = 3. * *p* ≤ 0.05, ** *p* ≤ 0.01 compared to each normalized control.

The effect of EGF on the cellular uptake of PS NPs by A431 cells was examined and the results are shown in Figure 2. The percentage of control cells containing NPs was approximately 30%, whereas the cellular uptake ratio of PS NPs by cells treated with 100 ng/mL of EGF increased to approximately 70%. EGF enhanced cellular uptake of PS NPs by dose dependent manor (data not shown), and 100 ng/mL of EGF showed higher enhancing than lower concentration of EGF. We confirmed the increase in cellular uptake by EGF and EGFR by adding anti-EGFR antibody, which blocks the binding of EGF to EGFR. We first tested the effect of anti-EGFR antibody (20 ng/mL) alone or in combination with EGF (100 ng/mL). Treatment with anti-EGFR antibody (20 ng/mL) alone had no significant effect on the uptake ratio of PS NPs by A431 cells, whereas treatment with a combination of EGF and anti-EGFR antibody significantly decreased the uptake ratio. Anti-EGFR antibody blocked cellular uptake by dose dependent manor (data not shown), and 20 ng/mL of anti-EGFR antibody showed higher blocking effects than lower concentration of antibody. The results suggested that the EGF-EGFR complex participates in cellular uptake triggered by an increase in EGF.

### 3.3. The Cellular Uptake of PS NPs by EGF Induction Occurs through Clathrin-Mediated Endocytosis

**Figure 3:**
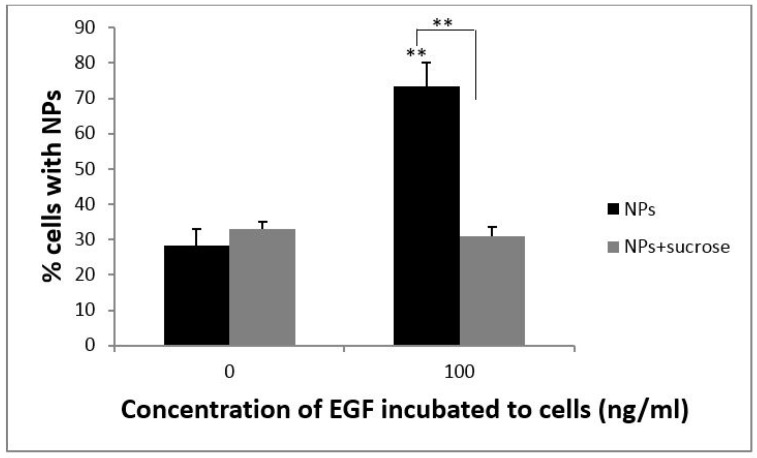
Effect of sucrose on cellular uptake efficiency of PS NPs. A431 cells were exposed to 10 µg/mL of PS NPs and 100 ng/mL EGF with (gray bars) or without (black bars) 0.45 M sucrose, at 37 °C for 24 h. The cellular uptake efficiency of NPs was normalized to that of untreated control cells. Mean values ± standard deviation, *n* = 3. ** *p* ≤ 0.01 compared to each normalized control.

Several cellular pathways could be involved in the process of internalizing NPs, such as phagocytosis and endocytosis. To further elucidate the mechanism of cellular uptake of PS NPs, cells were incubated in hyperosmotic sucrose solution to inhibit clathrin-mediated endocytosis. A431 cells were incubated with PS NPs with or without EGF (100 ng/mL) and with or without sucrose solution (0.45 M). As shown in Figure 3, the uptake ratio of PS NPs was significantly reduced in cells treated with sucrose and EGF (right gray bar), similar to that of cells not treated with EGF. These results showed that sucrose decreased the effect of EGF on cellular uptake of PS NPs. In contrast, in cells treated with sucrose but not EGF (left gray bar), the ratio of cellular uptake was similar to that of the control cells. This result indicated that PS NPs used different pathways to enter the cells in the presence or absence of EGF. In the presence of EGF, cellular uptake of PS NPs was via clathrin-mediated endocytosis, whereas, in the absence of EGF, uptake of PS NPs did not involve clathrin-mediated endocytosis. Consequently, EGF enhanced cellular uptake of PS NPs by clathrin-mediated endocytosis. Sucrose is known to disrupt the formation of clathrin-vesicles, and thus cellular uptake triggered by EGF was predominantly via endocytosis involving clathrin-coated vesicles. However, in the absence of EGF, NPs were taken up via another pathway, independent of clathrin-coated vesicles.

### 3.4. The Localization of EGFRs and PS NPs in A431 Cells with or without EGF

**Figure 4:**
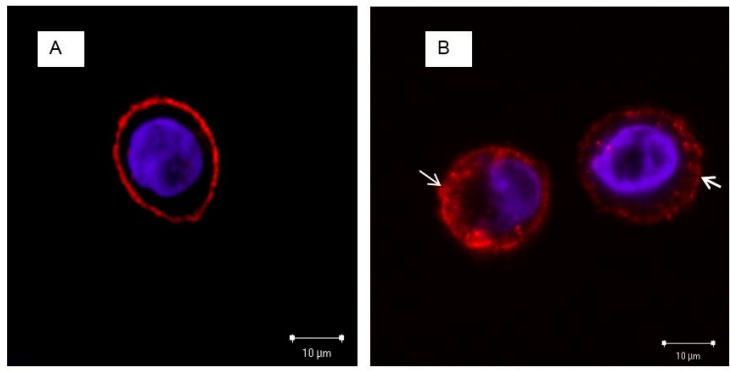

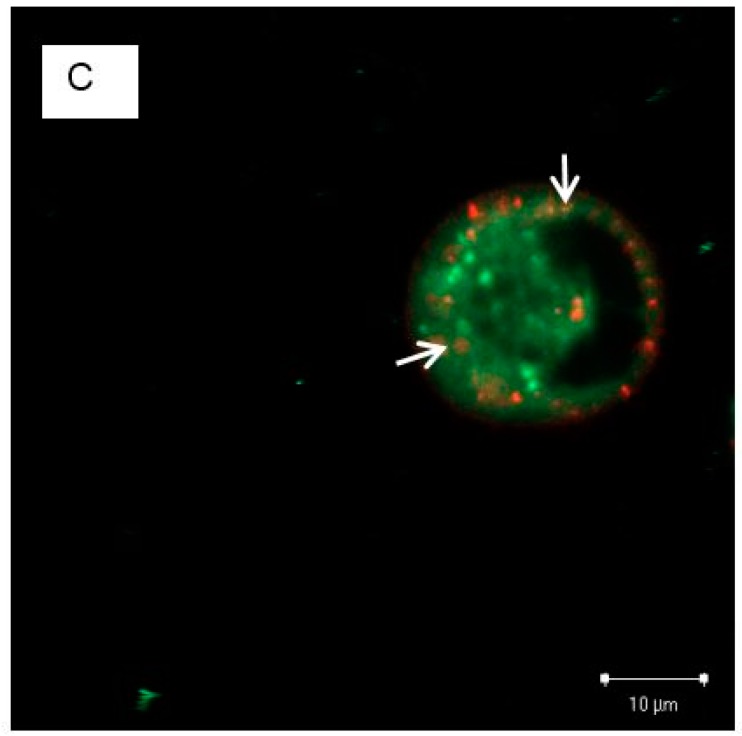
Localization of EGFR and PS NPs in A431 cells. Typical three-color merged confocal fluorescence microscopy images of A431 cells stained with: (**A**) anti-EGFR antibody; or exposed to: (**B**) 100 ng/mL EGF; or (**C**) 100 ng/mL EGF + 10 µg/mL NPs. Without EGF, EGFR distributed homogeneously on the cell membrane. In the presence of EGF, EGF induced slight aggregation of EGFRs. The presence of EGF and PS NPs resulted in co-localization of EGFRs and NPs in the cytoplasm. EGFRs are shown in red, NPs in green, and the cell nucleus (stained with DAPI) in blue.

We confirmed the role of EGF and EGFR in PS NP cellular uptake by localizing the EGFRs and PS NPs by confocal microscopy. The results indicated that without EGF, EGFRs were distributed homogeneously on the cell membrane (Figure 4A). In contrast, EGF induced changes in the localization of EGFRs: in the presence of 100 ng/mL EGF, EGFR aggregated slightly and formed several receptor clusters on the cell surface, and EGFR was also detected in the cytoplasm (Figure 4B). When cells were exposed to EGF and PS NPs, EGFR and PS NPs co-localized in the cytoplasm (Figure 4C), indicating that cellular uptake of PS NPs involves the binding of aggregated EGF–EGFR complexes and PS NPs. Sometime, labeled NPs had different character with none-labeled one. We could not deny fluorescent label affected to EGF-EGFR complexes.

## 4. Discussion and Conclusions

In this work, we studied the effects of EGF on cellular uptake and clarified the principal cellular uptake pathway of PS NPs. First, A431 cells were exposed to different concentrations of PS NPs and the cellular uptake ratio was determined using flow cytometry. The result showed that the uptake of PS NPs increased in a PS NP dose-dependent manner. Next, we also investigated the binding inhibition of EGF to EGFR by anti-EGFR antibody and observed that anti-EGFR antibody decreased the uptake efficiency of NPs into cells. Typically, after binding to a ligand such as EGF, EGFR forms homodimers or heterodimers with other members of the ErbB family of receptor tyrosine kinases (RTKs), thereby activating various downstream signaling pathways [25]. Anti-EGFR antibody would compete with EGF to bind to EGFR. This inhibition would reduce the number of receptor clusters and lead to decreased particle uptake efficiency. In addition, the effect of EGF on the internalization of PS NPs was examined. A previous study showed that at high concentration (≥0.3 nM), EGF inhibited the growth of A431 cells [1], and thus changes in the growth of cells caused by EGF could affect their ability to uptake particles. Therefore, in this study a high concentration of EGF (100 ng/mL) was used. The flow cytometry results revealed that a high dose of EGF enhanced PS NP uptake into the cells. To determine the cellular uptake pathway, we added sucrose, which is known to disrupt the formation of clathrin vesicles [26,27,28]. We observed that sucrose significantly decreased the uptake ratio of NPs when a high concentration of EGF was present, whereas no difference in uptake efficiency was observed without added EGF. These findings indicate that cellular uptake of PS NPs is enhanced by EGF, and PS NPs use different pathways to enter cells, dependent or independent of clathrin-mediated endocytosis, corresponding to the presence or absence of added EGF.

**Figure 5:**
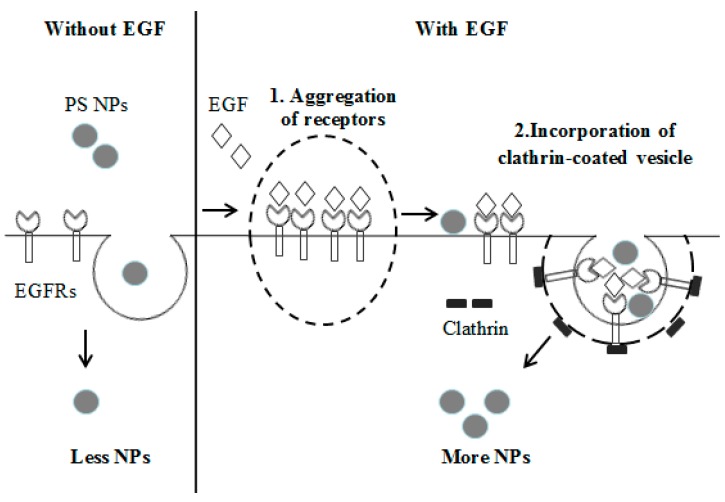
Hypothesis explaining the molecular mechanism for cellular uptake of PS NPs. When culture medium lacks EGF, most PS NPs pass through the cell membrane via the endocytosis pathway. In the presence of added EGF, the aggregation of EGFRs provides more space on the cell membrane, allowing NPs to enter the cells easier, and also NPs could combine with the EGF-EGFR complex, allowing their uptake into the cells through clathrin-mediated endocytosis.

Our hypothesis explaining the observed differences in uptake efficiency of PS NPs by A431 cells is shown in Figure 5. When cells are incubated with a high concentration of EGF, EGF induces EGFR aggregation and receptor clustering. Previous studies concluded that stimulation with 100 ng/mL EGF caused EGFR aggregation on the plasma membrane, followed by receptor internalization after longer stimulation times [29,30]. In the present study, we observed that PS NPs could combine with the EGF-EGFR complex and be internalized by A431 cells by clathrin-mediated endocytosis together with the EGF-EGFR complex, resulting in a large number of internalized PS NPs. In contrast, in the absence of EGF, few PS NPs would be internalized through the endocytosis pathway, independent of the EGF-EGFR complex. When the culture medium did not contain EGF, the EGFRs were distributed homogeneously on the cell surface (Figure 4A), whereas EGFRs slightly aggregated in the presence of a high concentration of EGF (Figure 4B). EGFRs and NPs co-localized in the cytoplasm of cells exposed to 100 ng/mL EGF and NPs (Figure 4C). Therefore, our results show that EGF enhances the cellular uptake of PS NPs by promoting the aggregation of EGFRs and by binding PS NPs to the EGF-EGFR complex via clathrin-mediated endocytosis. The other possibility is the EGF protein would associate with the NPs first, leading to their enhanced association with EGFR. To provef this hypothesis, direct evidence for the interaction between PS NPs and EGF-EGFR complex could be needed.
